# Comparative analysis of nuclear, chloroplast, and mitochondrial genomes of watermelon and melon provides evidence of gene transfer

**DOI:** 10.1038/s41598-020-80149-9

**Published:** 2021-01-15

**Authors:** Haonan Cui, Zhuo Ding, Qianglong Zhu, Yue Wu, Boyan Qiu, Peng Gao

**Affiliations:** 1 College of Horticulture and Landscape Architecture, Northeast Agricultural University, Harbin, 150030 Heilongjiang People’s Republic of China; 2 Key Laboratory of Biology and Genetic Improvement of Horticulture Crops (Northeast Region), Ministry of Agriculture, Harbin, 150030 Heilongjiang People’s Republic of China; 3 Department of Horticulture, College of Agronomy, Jiangxi Agricultural University, Nanchang, People’s Republic of China

**Keywords:** Genetics, Plant sciences

## Abstract

During plant evolution, there is genetic communication between organelle and nuclear genomes. A comparative analysis was performed on the organelle and nuclear genomes of the watermelon and melon. In the watermelon, chloroplast-derived sequences accounted for 7.6% of the total length of the mitochondrial genome. In the melon, chloroplast-derived sequences accounted for approximately 2.73% of the total mitochondrial genome. In watermelon and melon, the chloroplast-derived small-fragment sequences are either a subset of large-fragment sequences or appeared multiple times in the mitochondrial genome, indicating that these fragments may have undergone multiple independent migration integrations or emerged in the mitochondrial genome after migration, replication, and reorganization. There was no evidence of migration from the mitochondria to chloroplast genome. A sequence with a total length of about 73 kb (47%) in the watermelon chloroplast genome was homologous to a sequence of about 313 kb in the nuclear genome. About 33% of sequences in the watermelon mitochondrial genome was homologous with a 260 kb sequence in the nuclear genome. A sequence with a total length of about 38 kb (25%) in the melon chloroplast genome was homologous with 461 sequences in the nuclear genome, with a total length of about 301 kb. A 3.4 Mb sequence in the nuclear genome was homologous with a melon mitochondrial sequence. These results indicate that, during the evolution of watermelon and melon, a large amount of genetic material was exchanged between the nuclear genome and the two organelle genomes in the cytoplasm.

## Introduction

Watermelon (*Citrullus lanatus* [Thunb.] Matsum. Et Nakai) originated in Africa^1^, and Melon (*Cucumis melo* L.) originated in Africa^2^ or Asia^3^. Both are annual dicotyledonous plants of the genus Cucurbitaceae. Studies have shown that the chloroplast and mitochondrial genomes of watermelon and melon are related to important physiological activities, such as photosynthesis, respiration, cold tolerance, and sex differentiation^4,5^, and they are also useful for germplasm resource identification and phylogenetic research^3,6^.

Chloroplasts have bilayer membrane structures and are organelles particular to plant cells. The chloroplast genome of higher plants is generally between 115 and 165 kb, and the structure is highly conserved. The chloroplast genome of tobacco was the first to be sequenced in higher plants^7^; its genome comprises a single-loop double-stranded DNA molecule with a typical structure of four segments, each of which has a large single-copy (LSC) region, a small single-copy (SSC) region, and two inverted repeat (IR) regions (IRa and IRb)^8^. The LSC region in the chloroplast genomes of most plants is between 81 and 90 kb, the SSC region is between 18 and 20 kb, and the maximum variation in the length of the IR region is between 5 and 76 kb^9^. The LSC and SSC regions are separated by the two IR sequences. During the evolution of angiosperms, the order of these four parts of the chloroplast genome have remained almost unchanged.

Mitochondria are found in most cells and, like chloroplasts, are surrounded by a membrane bilayer. Compared with the smaller, conserved chloroplast genome, the plant mitochondrial genome has more complex structural features, usually comprised of a single double-stranded circular DNA molecule, such as in *Arabidopsis*^10^, watermelon, and zucchini^11^. Some mitochondrial genomes also exist as linear DNA molecules, such as that of rice^12^. In addition, multiple circular mitochondrial genomes have been found in a wide range of plants, such as wheat^13^, rape^14^, and cucumber^15^. Although the number of genes encoded by the mitochondrial genome varies from plant to plant, the types and sequences of the genes are very conserved. In fact, the evolution rate is the slowest and most conservative of the three sets of plant genomes, and the position and arrangement are quite different^16^.

In theory, there are six types of sequence migration or endogenous gene transfer in cells. That is, when a nuclear sequence is endogenously transferred into the mitochondria or chloroplasts, mitochondrial sequences are endogenously transferred to the nucleus or chloroplasts, and chloroplast sequences are endogenously transferred to the nucleus or mitochondria^17–24^. In general, plastid genomes are thought to be highly conservative and do not integrate much foreign genetic material^25^. Although, the mitochondrial genome of seed plants is thought to integrate sequences from chloroplasts, the nucleus^26–28^, and other mitochondria^29^.

The intergenic region accounts for a large proportion of the plant mitochondrial genome and is poorly conserved. It mainly includes foreign migrating fragments and chimeric sequences composed of repetitive segments mediated through recombination. The sequences of exogenous migrating fragments are mainly obtained from the chloroplast and nuclear genomes via horizontal gene transfer, but they may also be transferred from bacterial genomes, such as was found in the mitochondrial genome of cucumber^15^. Repetitive sequences are the most common and important sequences in the mitochondrial intergenic region. They are related to the mutation and recombination of the mitochondrial genome and play an important role in the evolution of plant mitochondrial genomes.

In this study, we compared sequence homologies between chloroplast, mitochondria, and nuclear genomes to estimate the amount of genetic material exchanged between the organelles and nuclei in watermelon and melon. In addition, we aimed to provide basic data for future related research into watermelon and melon nuclear–cytoplasm interactions.

## Materials and methods

### DNA isolation, genome sequencing and assembly of Watermelon and Melon

The watermelon cultivars W1-1 and melon cultivars MR-1 were planted in a plastic greenhouse at the Horticultural Station of the Northeast Agricultural University, Harbin, China (44° 04′ N, E125° 42′). And the individuals were using standard horticultural procedures (irrigation, hand-weeding, and pathogen prevention and control) typical of the climatic conditions in Harbin.

Young leaves without any damage of W1-1 and MR-1 were collected and quickly transferred into storage at − 80 °C until DNA extraction. Total DNA was extracted using the modified cetyl trimethyl ammonium bromide method^30^. The ultrasonically fragmented DNA samples were purified, distally repaired, ligated sequencing joint, then were chosen by 1% agarose gel electrophoresis to obtain a target insert size of 500–600 bp and purified for further analysis. PCR amplification was performed to construct a paired-read sequencing library. The DNA libraries were sequenced on an Illumina HiSeqTM 2500 platform (Biomarker, Beijing, China) to generate 125 base paired-end reads. The Re-Sequencing data of W1-1 and MR-1 were deposited in GenBank (https://www.ncbi.nlm.nih.gov/) under BioProject ID PRJNA682698.

### Alignment of paired-end reads to the reference sequence

We downloaded chloroplast and mitochondrial genomic data for the watermelon and melon from the NCBI database (Table 1), and we downloaded watermelon (97,103) genome v2^31^ and melon (DHL92) genome 3.5.1^32^ from the Cucurbitaceae genome website (http://cucurbitgenomics.org).

**Table 1:** GeneBank numbers for watermelon and melon organelle genomes.

	Mitochondrial genomes	Chloroplast genomes
Watermelon	NC_014043	NC_032008
Melon	MG947207	NC_015983
	MG947208	
	MG947209	

To obtain clean, high quality reads, the raw reads of all samples were filtered according to strict parameters as described previously^33^. After the reads were checked for quality, the cleaned reads of W1-1 and MR-1 were aligned to the reference genome using built-in BWA aligner (version 0.6.1-r104)^34^. Sequences with sequencing depth greater than 30 were selected as credible watermelon and melon nuclear genomes, chloroplast genomes and mitochondrial genomes for subsequent analysis.

### Comparative analysis of organelle and nuclear genomes

Genomic-scale sequence comparisons were carried out with LASTv7.1.4^35^ to map regions sharing similar sequences within a genome and to identify the sequences shared between chloroplast and mitochondrial genomes. The software HS-BLASTN v0.0.5^36^ and Tbtools v1.064^37^ were used to analyze the sequence comparisons between the organelle and nuclear genomes, with all software parameters set to default.

### Statistical analysis and graphics

All of the statistical analyses were performed using R v3.3.3^38^, and the figures were generated using CIRCUS^39^ and ggplot2^40^ in R.

## Results

### Re-sequencing and assembly of nuclear genomic and organelle genomes in watermelon and melon

A total of 101,667,347 clean reads were obtained from resequencing of watermelon cultivated material W1-1, totaling 15,250,102,050 bp. And 14,945,100,009 bp were spliced to the nuclear genome, 13,894,024 bp to the mitochondrial genome, and 5,434,804 bp to the chloroplast genome by comparison with the reference genome, with an average sequencing depth of 34. A total of 75,866,316 clean reads were obtained from resequencing of melon cultivated material MR-1, totaling 11,379,947,400 bp. And 10,355,752,134 bp were spliced to the nuclear genome, 91,285,780 bp to the mitochondrial genome, and 5,680,510 bp to the chloroplast genome by comparison with the reference genome, with an average sequencing depth of 30. Sequences with sequencing depth greater than 30 were selected as credible watermelon and melon nuclear genomes, chloroplast genomes and mitochondrial genomes for subsequent analysis.

### Comparison of chloroplast and mitochondrial genome characteristics of watermelon

Although the watermelon mitochondrial genome is about 2.4 times the size of the chloroplast genome (Table 2), the number of protein-coding genes was found to be only about one-half that of the chloroplast genome. The protein-coding genes of the chloroplast genome accounted for about 50% of the total genome length, while the protein-coding genes of the mitochondrial genome only comprised 8.58% of the genome. The 26 introns of the watermelon chloroplast genome were distributed among 18 genes, with a single gene having no more than two introns. The 24 introns of the watermelon mitochondria genome were distributed among 11 genes, and some genes, such as the *nad* gene family, *nad2*, *nad5*, and *nad7*, had up to four introns. The chloroplast genome of watermelon encodes a set of tRNA genes that it can use to synthesize its own proteins, whereas the mitochondrial genome lacks the tRNA to recognize some amino acids and needs to import tRNA from the cytoplasm for protein synthesis.

**Table 2:** The basic features of chloroplast and mitochondrial genomes in *C. lanatus.*

Features	Chloroplast	Mitochondrion
Genome size (bp)	156,906	379,236
Chromosome number	1	1
GC (%)	37.18	45.08
Gene number	130	63
Protein-coding gene number	86	39
Protein-coding sequence (bp)	78,468 (50.01%)	32,553 (8.58%)
Genes with introns (cis/trans)	18/2	11/3
Intron no. (cis/trans)	24/2	20/4
Cis-splicing intron (bp)	19,599 (12.59%)	32,735 (8.63%)
tRNA number	37 (30)	18 (15)
tRNA sequence (bp)	2807 (1.80%)	1454 (0.38%)
rRNA number	8 (4)	3 (3)
rRNA sequence (bp)	9394 (5.98%)	5145 (1.35%)

The count excluding the gene duplicates. The note is applicable only to the numbers mentioned in brackets for features tRNA number and rRNA number.

### Sequence migration between chloroplast and mitochondrial genomes of watermelon

**Figure 1:**
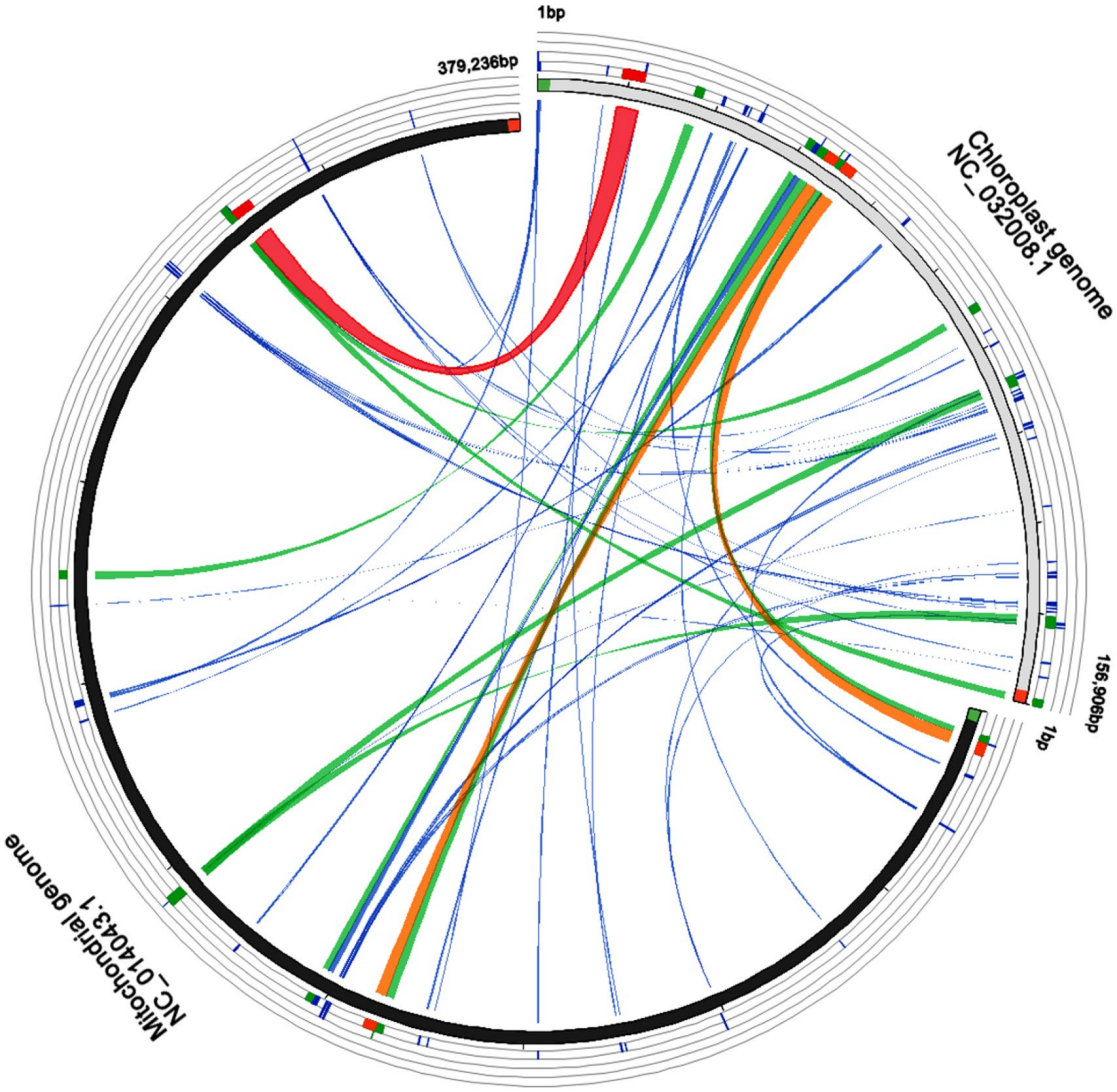
The transfer of sequences from chloroplast to mitochondrial genomes in *C. lanatus.*

This study found that, in the mitochondrial genome of the watermelon, there were 34 chloroplast-derived sequences with a length of more than 50 bp, a similarity of no less than 80% (Fig. 1; Table 3), and a total length of 28,703 bp, accounting for about 7.6% of the mitochondria genome. The homologous sequences in the mitochondrial genome of the watermelon made up 18.3% of the total length of the chloroplast genome, in which there were 25 sequence fragments with a length of more than 100 bp (Supplementary Table S1). Among these fragments, 12 fragments were 100–500 bp in length, 2 fragments were 500–1000 bp, and 11 fragments were longer than 1 kb. With regards to distribution, 16 were from the chloroplast genomic LSC region, 9 were from the IR region, and there were no fragments from the SSC region. The longest fragment, of 3822 bp, originated from the LSC region and included four genes in the chloroplast. The second and third longest migration fragments were about 3646 bp and 3626 bp, all from the LSC region. Furthermore, it was found that some small fragment sequences derived from chloroplasts were a subset of large fragment sequences or appeared multiple times in the mitochondrial genome, such as the sequence fragments with the sequence numbers 18 and 19, indicating that these fragments underwent multiple independent migration integrations or that replication and recombination occurred within the mitochondrial genome after migration.

**Table 3:** Summary of the transfer of sequences from chloroplast to mitochondrial genomes in *C. lanatus.*

Fragment length (bp)	Number	Total length (bp)	PTM (%)	PTC (%)
50–100	9	623	0.2	0.4
100–500	12	3250	0.8	2.1
500–1000	2	1726	0.5	1.1
1000<	11	23,104	6.1	14.7
Total	34	28,703	7.6	18.3

*PTM* Percentage of transferred sequences in mitochondrial genome, *PTC* percentage of transferred sequences in chloroplast genome.

### Sequence migration between chloroplast, mitochondrial, and nuclear genomes of watermelon

**Figure 2:**
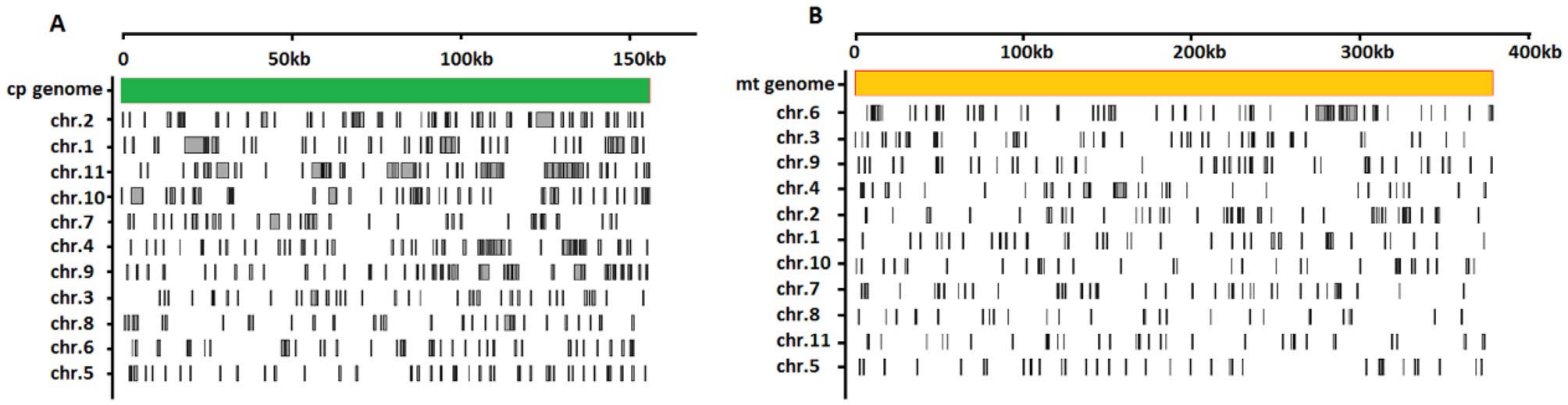
Distribution of homologous genomic sequences between the chloroplast, mitochondria, and nucleus in *C. lanatus.* (**A**) Indicates comparisons between chloroplast genome and nuclear genome; (**B**) indicates comparisons between the mitochondrial and nuclear genomes.

We analyzed the alignment of sequences between the chloroplast, mitochondrial, and nuclear genomes (Fig. 2). There were 137 sequences in the watermelon chloroplast genome with a total length of about 73 kb (47%) and 567 sequences in the nuclear genome with a total length of about 313 kb. The length of the 137 sequences in the chloroplast genome ranged from 222 to 4808 bp, with an average length of 532 bp. The 567 homologous fragments in the nuclear genome were unevenly distributed on 11 watermelon chromosomes. The chloroplast genome had the most homology with chromosome 2 and the least homology with chromosome 5. On average, each homologous chloroplast sequence was similar to four different regions in the nuclear genome. A sequence of about 125 kb (33%) in the watermelon mitochondrial genome was homologous to a 260 kb sequence in the nuclear genome. The mitochondrial genome had the most sequence homology with chromosome 6, and 263 fragments in the mitochondria corresponded to 520 fragments in the nuclear genome with lengths ranging from 261 to 4246 bp and an average length of 474 bp. On average, each mitochondrial fragment was homologous to two different regions on the nuclear genome. These results indicate that, during the evolution of watermelon plants, the nuclear and two organelle genomes in the cytoplasm exchanged a large amount of genetic material.

### Comparison of chloroplast and mitochondrial genomic characteristics of melon

In the melon, the mitochondrial genome is about 18.7 times the size of the chloroplast genome, but there are less than half the number of protein-coding genes in the mitochondria compared with the chloroplast genome. Furthermore, the mitochondrial protein-coding gene sequences in the melon account for only 1.28% of the entire mitochondrial genome. In contrast, the chloroplast protein coding gene sequences account for 51.62% of the entire chloroplast genome (Table 4). The 26 introns of the melon chloroplast are distributed among 18 genes, and a single gene has no more than two introns. The 22 introns of melon mitochondria are distributed among 10 genes. In some genes, such as *nad1*, *nad2*, and *nad5*, there were as many as four introns. Due to frequent recombination of the internal structure of the genome, the number of introns in the melon mitochondrial genome has increased by trans-splicing.

**Table 4:** The basic features of chloroplast and mitochondrial genomes in *C. melo.*

Features	Chloroplast	Mitochondrion
Genome size (bp)	156,017	2,906,673
Chromosome number	1	3
GC (%)	36.9	44.77
Gene number	132	88
Protein genes number	87	40
Protein genes sequence (bp)	80,433 (51.62%)	37,140 (1.28%)
Genes with intron (cis/trans)	18/1	10/3
Intron no. (cis/trans)	24/2	17/5
Cis-splicing intron (bp)	20,286 (13.02%)	40,886 (1.41%)
tRNA number (type)	37 (30)	40 (17)
tRNA sequence (bp)	2820 (1.81%)	3284 (0.11%)
rRNA number (type)	8 (4)	8 (3)
rRNA sequence (bp)	10,620 (6.81%)	5815 (0.20)

The count excluding the gene duplicates. The note is applicable only to the numbers mentioned in brackets for features tRNA number and rRNA number.

### Chloroplast and mitochondrial genomic sequence transfer in melon

**Figure 3:**
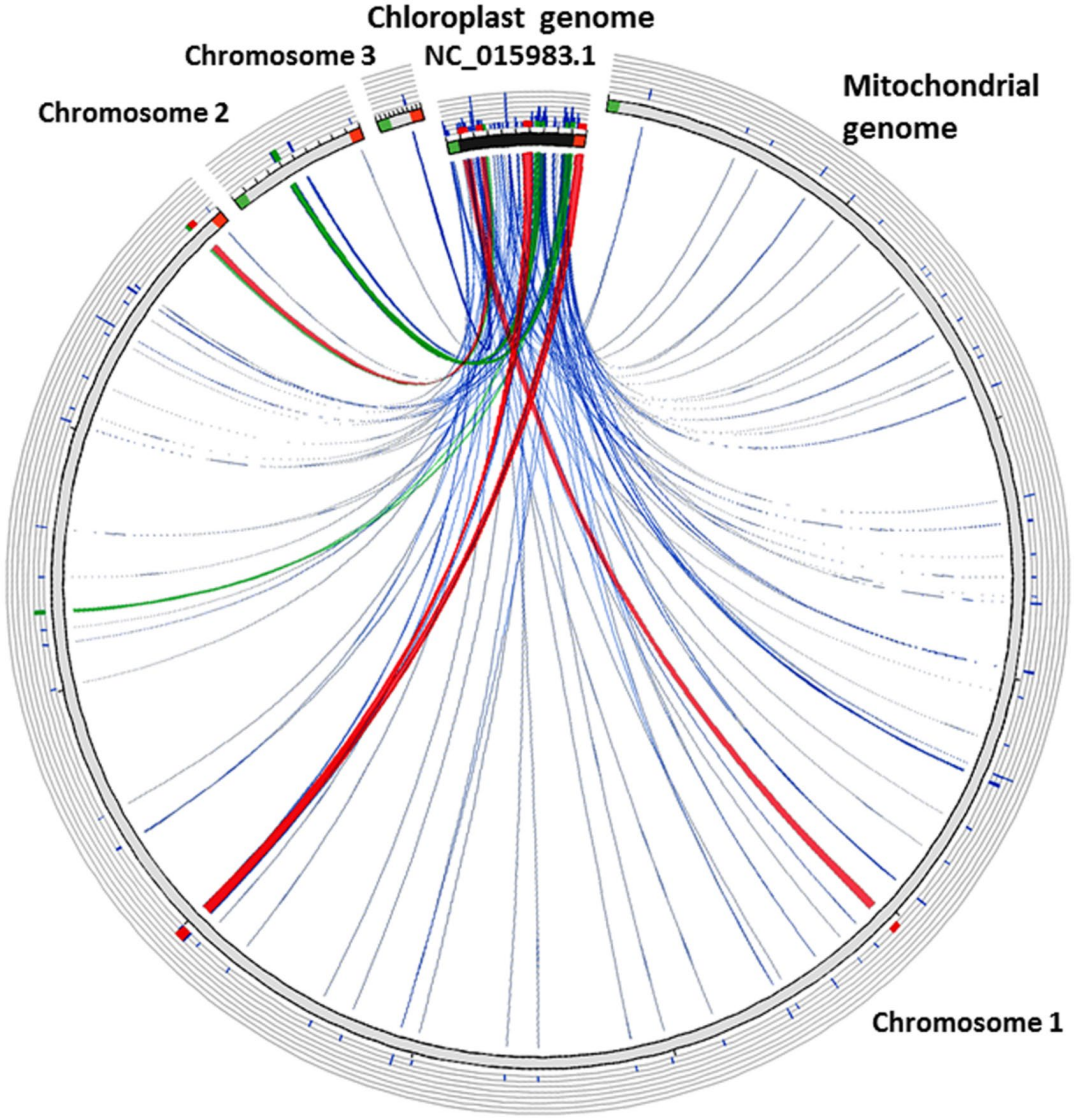
Transfer of sequences from chloroplast to mitochondrial genomes in *C. melo.*

In this study, we analyzed the sequences in the melon mitochondrial genome that have migrated from the chloroplast genome and found 91 chloroplast-derived sequences with a length of more than 50 bp and a similarity of no less than 80% (Fig. 3; Table 5). The total length of migrated sequences was 79,4630 bp, about 2.73% and 50.99% of the full length of the mitochondrial and chloroplast genomes, respectively. Among the migration sequences, 33 fragments were shorter than 500 bp, 58 were longer than 100 bp, 25 were longer than 500 bp, and 19 fragments were longer than 1 kb (Supplementary Table S2). Among these fragments, 20 were from the chloroplast genome LSC region, 34 were from the IR regions, and 4 were from the SSC region, indicating that most of the mitochondrial sequences originating from the chloroplast genome came from the repeat regions. The second and third longest migration fragments were approximately 9145 bp and 8968 bp, from the LSC and IR regions, respectively. At the same time, it was found that some small fragment sequences derived from chloroplasts were a subset of large fragment sequences or had appeared multiple times in the mitochondrial genome, such as sequence fragments with sequence numbers 34–37 and 42–45, indicating that these fragments emerged from multiple independent migration and integration events, or replication events, followed by recombination^11^.

**Table 5:** Summary of the transfer of sequences from chloroplast to mitochondrial genomes in *C. melo.*

Fragment length (bp)	Number	Total length (bp)	PTM (%)	PTC (%)
50–100	33	1749	0.06	1.12
100–500	33	6860	0.24	4.41
500–1000	6	4397	0.15	2.82
1000<	19	66,457	2.28	42.65
Total	91	79,463	2.73	50.99

*PTM* Percentage of transferred sequences in mitochondrial genome, *PTC* percentage of transferred sequences in chloroplast genome.

### Sequence transfer between chloroplast, mitochondrial, and nuclear genomes of melon

**Figure 4:**
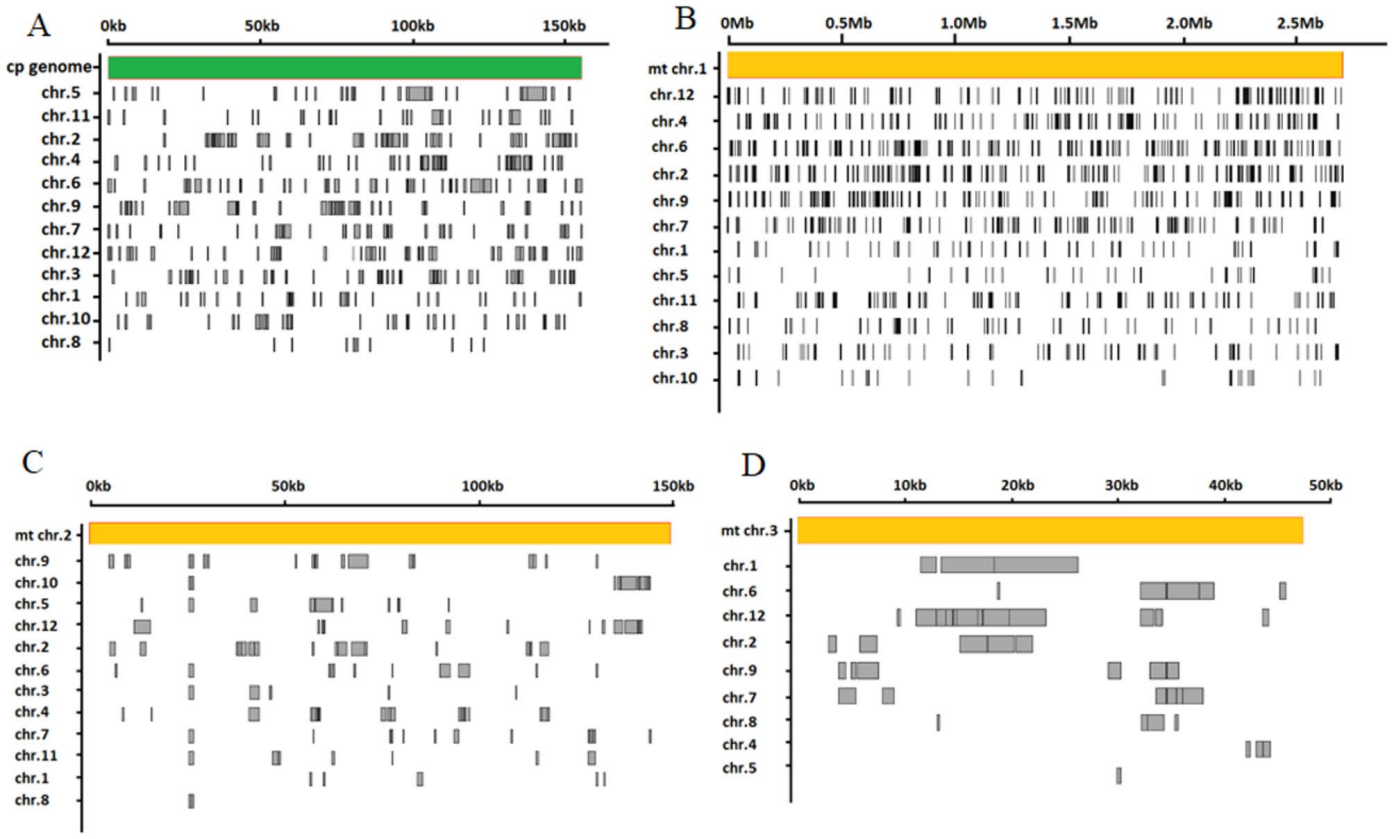
Distribution of homologous sequences within the nuclear, chloroplast, and mitochondrial genomes of *C. melo.* (**A**) indicates comparisons between chloroplast and nuclear genomes; (**B**–**D**) indicate comparisons between mitochondrial and nuclear genomes.

Analysis of sequence homologies between the chloroplast, mitochondrial, and nuclear genomes in melon (Fig. 4) showed that there were 108 sequences in the melon chloroplast genome with a total length of approximately 38 kb (25%). In total, 461 sequences with a total length of about 301 kb showed homologous relationships. The 108 sequences in the chloroplast genome were all 204 bp to 1039 bp in length, with an average length of 363 bp. The 461 homologous fragments in the nuclear genome were unevenly distributed throughout the entire melon genome. Among these, chromosome 5 had the most homologous sequences in common with the chloroplast genome, while chromosome 8 had the least homology. On average, each homologous sequence in the chloroplast was similar to four different regions in the nuclear genome; approximately 1413 kb (48.62%) of sequence in the melon mitochondrial genome was homologous to 3.4 Mb of the nuclear genome. While 1048 fragments in the mitochondria corresponded to 3391 fragments on the nuclear genome. On average, each mitochondrial fragment was homologous to three different regions in the nuclear genome. The 1,048 homologous sequences in the mitochondrial genome ranged from 214 bp to 6120 bp, with an average length of 941 bp.

## Discussion

Sequences that have migrated from chloroplast genomes have been found in the mitochondrial genomes of all plants sequenced so far, and this part of the genome usually accounts for 1–12% of the total length^41^. It is considered almost impossible for sequences from the plant mitochondrial genome to transfer into the chloroplast genome^42^. The total length of homologous sequences between the melon chloroplast and mitochondrial genomes is approximately 79 kb, about 2.73% of the whole mitochondrial genome, indicating that these sequences may be derived from the chloroplast. Like that of other angiosperms^43^, the chloroplast genome of the watermelon is relatively conservative and does not accept the integration of foreign DNA fragments. However, the mitochondrial genome contains a large number of sequences that have migrated from the chloroplast genome^44^. The mitochondrial genome of watermelon contains fewer homologous chloroplast genomic sequences, but mitochondria of the zucchini contain 113 kb of transferred chloroplast genomic sequences, which is approximately 80% of the total length of the chloroplast genome of the Cucurbitaceae plant^11^. Chloroplast genome fragments usually carry some protein-encoding genes during transfer to the mitochondrial genome, but these genes become non-functional pseudogenes after integration, which may be caused by genomic sequence reorganization^45^. Our analysis of chloroplast migration sequences in the mitochondrial genomes of watermelon and melon supports this conclusion.

Some non-protein-encoding genes, such as tRNA, still perform normal functions after they are transferred to the mitochondrial genome^46^. Compared with the melon, the watermelon’s smaller mitochondrial genome contains about 23 kb of migrated chloroplast sequences, and the zucchini mitochondrial genome contains about 113 kb of chloroplast sequences, indicating that there is no association between the size of the mitochondrial genome and the amount of chloroplast migration sequences. In this study, we found that the watermelon and melon mitochondrial genomes contain 3 and 17 chloroplast-derived tRNAs with complete structures, respectively, and we believe they may have normal transport functions. This characteristic may be a characteristic of higher plant mitochondria during evolution. At present, there are still many mysteries about the sequence migration mechanisms between the organelle genomes in the watermelon and melon and the expression patterns of genes carried by the migrated fragments. However, we believe that further development of the watermelon and melon whole genome project will help in the analysis of these problems.

In addition to the horizontal transfer of gene sequences between chloroplasts and mitochondria, there has also been a rich exchange of genetic material between the two organelle genomes and the nuclear genome. The mitochondrial and nuclear genomes can transfer DNA sequences in both directions. However, most studies have found that the chloroplast genome only transfers DNA sequences to the nuclear and mitochondrial genomes, not to other chloroplasts, and they tend not to integrate foreign DNA^47,48^. By analyzing the sequence homologies between the chloroplast, mitochondrial, and nuclear genomes, we can estimate the amount of genetic material exchanged between the organelles and the nucleus. Furthermore, the analysis can provide basic data for related research on nuclear-plastid interactions in the watermelon and melon.

## Conclusion

The results indicated that, during the evolution of the watermelon and melon, some small fragments derived from chloroplasts may have undergone multiple independent migrations and integrations, or that replication and recombination occurred after integration, within the mitochondrial genome. This study found that a large amount of genetic material has been exchanged between the genomes. By analyzing the sequence homologies between the chloroplast, mitochondrial, and nuclear genomes, we can estimate the amount of genetic material exchanged between the organelles and the nucleus. Furthermore, the analysis can provide basic data for related research on nuclear-plasmon interactions in the watermelon and melon.

## Supplementary Information

Supplementary Information.

## Data Availability

The re-sequencing data of W1-1 and MR-1 were deposited in GenBank (https://www.ncbi.nlm.nih.gov/) under BioProject ID PRJNA682698.
