# Supplementary material for: Comparative analysis of nuclear, chloroplast, and mitochondrial genomes of watermelon and melon provides evidence of gene transfer
Source: Sci Rep. 2021 Jan 15;11:1595. doi: 10.1038/s41598-020-80149-9 (PMC7811005; doi:10.1038/s41598-020-80149-9)
Supplement: Supplementary file 1 — Supplementary Information. [file 41598_2020_80149_MOESM1_ESM.pdf]

Comparative Analysis of Nuclear, Chloroplast, and Mitochondrial Genomes of Watermelon and Melon Provides Evidence of Gene Transfer

Haonan Cui[1,2], Zhuo Ding[1,2], Qianglong Zhu[3], Yue Wu[1,2], Boyan Qiu[1,2], Peng Gao [1,2*]

[1] College of Horticulture and Landscape Architecture, Northeast Agricultural University, Harbin, Heilongjiang 150030, China

[2] Key Laboratory of Biology and Genetic Improvement of Horticulture Crops (Northeast Region), Ministry of Agriculture, Harbin, Heilongjiang 150030, China

[3] Department of Horticulture, College of Agronomy, Jiangxi Agricultural University, Nanchang, P.R. China

*Correspondence: gaopeng_neau@163.com (P.G.); Tel.: 86-0451-55191261

Table S1. Chloroplast-derived sequences in *C. lanatus* mitochondrial genome (>100 bp)

Order	CpS[1](bp)	MtS[2](bp)	Length(bp)	Percentage identity (%)	Expect	Region	Genes
1	1	322,605	208	97.62	4E-98	LSC	*trnH-GTG*
2	200	224,147	377	97.88	0	LSC	*psbA**
3	14,467	322,800	3822	98.56	0	LSC	*atpH; atpI; rps2; rpoC2**
4	18,140	110,906	149	98	1E-68	LSC	*rpoC2**
5	26,967	248,777	1522	97.31	0	LSC	*rpoB**
6	31,841	170,250	353	81.37	7E-71	LSC	*trnD-GTC*
7	35,746	12,371	399	97.51	0	LSC	*psbC**
8	38,720	220,926	190	97.38	5E-87	LSC	*rps14**
9	38,910	132,920	230	96.54	3E-104	LSC	*rps14**
10	47,635	154,871	1147	98.35	0	LSC	*rps4*; trnT-TGT*
11	48,836	153,722	1034	93.95	0	LSC	*trnL-TAA*
12	49,856	139,713	3646	98.22	0	LSC	*trnF-GAA; ndhJ; ndhK; ndhC*
13	51,447	141,272	2037	98.25	0	LSC	*ndh*; ndhK; ndhC*
14	53,461	4,258	3626	95.54	0	LSC	*trnV-TAC; trnM-CAT; atpE; atpB*
15	68,844	224,414	348	93.71	1E-147	LSC	*trnP-TGG*
16	86,776	322,659	1319	99.85	0	LSC	*rpl2**
17	94,252	338,630	138	91.55	4E-48	IR	*ycf2**
18	100,293	183,916	1851	98.87	0	IR	*rps7; ycf15**
19	100,859	22,368	360	96.95	1E-172	IR	-
20	103,440	308,801	863	74.64	1E-83	IR	*rrn16**
21	139,449	307,943	863	74.64	1E-83	IR	*rrn16**
22	141,608	185,776	1,851	98.87	0	IR	*rps12**
23	142,533	22,008	360	96.95	1E-172	IR	-
24	149,362	338,770	138	91.55	4E-48	IR	-
25	155,657	321,340	1,249	99.84	0	IR	-

[1] CpS: Start loci of homologous sequence in chloroplast genome;

[2] MtS: Start loci of homologous sequence in mitochondrial genome.

[*]: There is this gene located in the derived sequence.

Table S2. Chloroplast-derived sequences in *C. melo* mitochondrial genome (>100 bp)

Order	CpS[1](bp)	MtS[2](bp)	Length (bp)	Percentage identity (%)	Expect	Region	Genes
1	1	chr.1:1,895,520	1172	90.62	0	LSC	*trnH-GTG; psbA**
2	276	chr.1:1,997,750	897	90.61	0	LSC	*psbA**
3	3960	chr.1:1,304,144	263	89.93	2E-88	LSC	-
4	14128	chr.1:2,595,818	816	97.68	0	LSC	*atpH*
5	14932	chr.1:1,090,875	9145	96.77	0	LSC	*atpI; rps2; rpoC2**
6	17511	chr.1:1,221,353	111	94.64	1E-41	LSC	*rpoC2**
7	20598	chr.3:33,387	399	85.86	1E-111	LSC	*rpoC2**
8	21060	chr.3:32,885	590	76.87	5E-70	LSC	*rpoC2**
9	22811	chr.1:1,871,718	103	88.99	3E-28	LSC	-
10	34725	chr.1:2,683,077	9616	98.88	0	LSC	*psbD; psbC; trnS-TGA; psbZ; trnG-GCC; trnfM-CAT; rps14; psaB; psaA*
11	37998	chr.1:1,121,210	151	83.33	9E-28	LSC	*psbZ**
12	40869	chr.1:1,011,729	234	79.45	2E-34	LSC	*psaB**
13	50325	chr.1:2,215,925	274	88.13	7E-84	LSC	*trnF-GAA*
14	55108	chr.1:172,852	226	89.04	4E-71	LSC	*atpB**
15	58835	chr.1:707,452	155	81.48	6E-25	LSC	-
16	60435	chr.1:2,701,421	177	82.89	2E-34	LSC	*accD**
17	69519	chr.1:650,303	133	92.54	1E-45	LSC	*rpl33**
18	78010	chr.1:1,359,278	368	95.12	8E-163	LSC	*petB**

19	78340	chr.1:971,097	120	90.91	3E-38	LSC	-
20	84083	chr.1:405,896	183	82.61	9E-33	LSC	-
21	86395	chr.1:1,895,461	8968	97.22	0	IR	*rpl2; rpl23; trnI-CAT; ycf2*
22	95352	chr.1:1,886,176	1000	94.92	0	IR	*ycf2*; trnL-CAA*
23	96346	chr.1:1,885,072	188	94.74	9E-78	IR	*ndhB**
24	99477	chr.2:62,905	395	90.44	2E-143	IR	*rps7**
25	99885	chr.2:62,428	4269	97.17	0	IR	*rps12*; ycf15; trnV-GAC; rrn16S; trnI-GAT*
26	100503	chr.1:232,059	125	84.25	3E-23	IR	-
27	102430	chr.1:734,116	242	85.77	2E-59	IR	*rrn16S*
28	102549	chr.1:803,752	732	75.39	7E-79	IR	*rrn16S*
29	104148	chr.2:58,159	1267	99.37	0	IR	*trnI-GAT*; trnA-TGC*
30	105430	chr.2:56,841	251	94.09	2E-104	IR	*trnA-TGC*; rrn23S**
31	105689	chr.1:1,057,870	1170	92.93	0	IR	*trnA-TGC*; rrn23S**
32	106502	chr.1:2,250,356	2994	96.22	0	IR	*rrn23S*; rrn4.5S; rrn5S**
33	108042	chr.1:1,231,985	180	81.77	3E-28	IR	*rrn23S**
34	109531	chr.1:921,001	364	89.54	3E-127	IR	*trnR-ACG*
35	109531	chr.2:78,638	364	89.54	3E-127	IR	*trnR-ACG*
36	109910	chr.1: 920,580	1797	91.77	0	IR	*trnN-GTT; ycf1**
37	109910	chr.2:78,217	1797	91.88	0	IR	*trnN-GTT; ycf2**
38	118409	chr.1:2,430,269	101	83.81	4E-16	SSC	*ndhD**
39	119717	chr.1:477,813	161	87.65	2E-44	SSC	*ndhE**

40	121186	chr.1:509,959	630	80.72	1E-120	SSC	*ndhI*; ndhA**
41	125063	chr.1:1,624,359	211	91.04	2E-74	SSC	*rps15**
42	130469	chr.1:918,805	1797	91.77	0	IR	*ycf1*; trnN-GTT*
43	130469	chr.2:76,440	1797	91.88	0	IR	*ycf2*; trnN-GTT*
44	132281	chr.1:920,636	364	89.54	3E-127	IR	*trnR-ACG*
45	132281	chr.2:78,273	364	89.54	3E-127	IR	*trnR-ACG*
46	132680	chr.1:2,247,363	2994	96.22	0	IR	*rrn5S; rrn4.5S; rrn23S**
47	133954	chr.1:1,232,141	180	81.77	3E-28	IR	*rrn23S**
48	135317	chr.1:1,059,026	1170	92.93	0	IR	*rrn23S*; trnA-TGC*
49	136495	chr.2:56,590	251	94.09	2E-104	IR	*trnA-TGC**
50	136761	chr.2:56,892	1267	99.37	0	IR	*trnA-TGC*; trnI-GAT**
51	138022	chr.2:58,189	4269	97.17	0	IR	*rrn16S; trnV-GAC; ycf15; rps12**
52	138895	chr.1:803,024	732	75.39	7E-79	IR	*rrn16S**
53	139504	chr.1:734,334	242	85.77	2E-59	IR	*rrn16S**
54	141548	chr.1:231,943	125	84.25	3E-23	IR	-
55	142304	chr.2:62,514	395	90.44	2E-143	IR	*rps7**
56	145642	chr.1:1,884,884	188	94.74	9E-78	IR	-
57	145824	chr.1:1,885,185	1000	94.92	0	IR	*trnL-CAA*
58	146813	chr.1:1,886,509	8968	97.22	0	IR	*trnI-CAT; rpl23; rpl2*

[1] CpS: Start loci of homologous sequence in chloroplast genome;

[2] MtS: Start loci of homologous sequence in mitochondrial genome.

[*]: There is this gene located in the derived sequence.
